# Surgical and postoperative evaluations of rectal adenomas excised with a rigid proctoscope^1^

**DOI:** 10.1590/s0102-865020200080000007

**Published:** 2020-09-18

**Authors:** Roberta Denise Alkmin Lopes de Lima, Rogério Serafim Parra, Marley Ribeiro Feitosa, Omar Feres, José Joaquim Ribeiro da Rocha

**Affiliations:** I Fellow PhD degree, Postgraduate Program in Surgical Clinic, Division of Coloproctology, Department of Anatomy and Surgery, Faculdade de Medicina de Ribeirão Preto, Universidade de São Paulo (FMRP-USP), Ribeirao Preto-SP, Brazil. Substantive scientific and intellectual contributions; conception and design of the study; acquisition, analysis and interpretation of data; manuscript preparation, final approval.; II PhD, Division of Coloproctology, Department of Anatomy and Surgery, FMRP-USP, Ribeirao Preto-SP, Brazil. Substantive scientific and intellectual contributions, manuscript writing, critical revision, final approval.; III PhD, Division of Coloproctology, Department of Anatomy and Surgery, FMRP-USP, Ribeirao Preto-SP, Brazil. Substantive scientific and intellectual contributions, analysis and interpretation of data, statistics analysis, final approval.; IV PhD, Associated Professor, Division of Coloproctology, Department of Anatomy and Surgery, FMRP-USP, Ribeirao Preto-SP, Brazil. Substantive scientific and intellectual contributions, analysis and interpretation of data, critical revision, final approval.; V PhD, Associated Professor, Head, Division of Coloproctology, Department of Anatomy and Surgery, FMRP-USP, Ribeirao Preto-SP, Brazil. Substantive scientific and intellectual contributions; conception and design of the study; acquisition, analysis and interpretation of data; critical revision, final approval.

**Keywords:** Adenomatous Polyps, Rectum, Transanal Endoscopic Surgery, Proctoscopes

## Abstract

**Purpose:**

This study presents the surgical and postoperative results achieved with a rigid proctoscope using the transanal endoscopic technique to excise rectal adenomas. The results are compared to the results obtained with other currently employed transanal techniques.

**Methods:**

We investigated the medical records of patients who underwent transanal endoscopic operations from April 2000 to June 2018 at two tertiary referral centers for colorectal cancer.

**Results:**

This study included 99 patients. The mean age was 65.3 ± 13.3 years. The average size of the adenomas was 4.6 ± 2.3 cm, and their average distance to the anal border was 5.6 ± 3.3 cm. The average operative time was 65.3 ± 41.7 min. In 48.5% of the operations, the specimen was fragmented, and in 59.6% of the cases, the microscopic margins were free. The rates of postoperative complications and relapse were 5% and 19%, respectively. The mean follow-up was 80 ± 61.5 months.

**Conclusions:**

The described proctoscope proved to be a viable technique with results similar to other techniques, with the advantage that it allowed greater accessibility for surgeons. Therefore, its use could be implemented and become widespread in surgical practice.

## Introduction

Colorectal cancer (CRC) is the third most common cancer in men and the second most common cancer in women worldwide and in 25% of cases are located in the rectum. Due to its slow progression from detectable precancerous lesions and to the much better prognosis of patients diagnosed at early stages, the potential for reducing the burden of the disease by early detection is significant^1,2^. Adenoma is the most important type of polyp because it is directly correlated with CRC and originates from the colorectal mucosa, accounting for approximately 70% of all polyps^3,4^.

Due to the malignant potential of rectal adenoma, excision is indicated, and the initial treatment consists of endoscopic removal during the diagnostic procedure^5^. When the rectal polyp size and/or location limit its resection by colonoscopy, surgeries are performed. In recent decades, transanal resection techniques have increasingly replaced invasive surgery in the treatment of premalignant rectal injury^6^.

Transanal endoscopic surgery (TES) can be performed by introducing different devices into the anal canal. TES techniques include transanal endoscopic microsurgery (TEM), transanal endoscopic operation (TEO), and transanal minimally invasive surgery (TAMIS), among other less well-known techniques. TEM consists of complex equipment and a beveled rectoscope, which is placed in the anus and forms an airtight seal to allow for insufflation of the rectum. Thus, very low tumors (<5 cm from the anal verge) are not visualized adequately with this procedure. The major disadvantages of TEM are the expense of the rectoscope and the learning curve associated with its use^7^. TEO appeared as a simpler system with a shorter learning curve, but a 3D optical system was not used, as in TEM^8^.

TAMIS appeared as an alternative to the more expensive system used for TEM and consists of using a single portal combined with common laparoscopic instruments. However, it is limited by the fact that the rectoscope cannot be mobilized at the injury site. Thus, rectal lesions located behind a rectal valve can be more difficult to access and remove. In addition, assistance is required to hold and manipulate the laparoscope during the procedure. The limitation for low injuries is the anal margin itself; however, system insufflation can be compromised for tumors less than 4 cm from the anal margin (9). When a decision about the surgical technique is being made, not only the lesion but also the patient’s age and comorbidities and their family’s as well as their own choices must be considered. Many techniques require a high level of surgeon expertise and experience.

Considering the experience of several hospital centers, the economic feasibility of acquiring materials, and the technical feasibility of staff training and experience with complex methods, performing a simpler technique with the resources that are available at institutions is relevant to excise selected rectal adenomas. This study presents the surgical and postoperative results achieved with a transanal endoscopic technique that was used to excise rectal adenomas with a rigid surgical proctoscope. The results are compared to the results obtained with other currently employed transanal techniques.

## Methods

This study was approved by the Research Ethics Committee of HCFMRP-USP (CAAE: 79769017.1.0000. 5440; opinion number: 2.427.871) on 12 November 2017. All procedures were in accordance with the institutional and national ethical standards of the responsible committee on human experimentation and with the 1964 Helsinki declaration and its later amendments or comparable ethical standards.

### *Study design*

A retrospective analysis of a prospectively collected database was conducted in the databases of two referral centers in the state of Sao Paulo, city of Ribeirao Preto, consisting of patients who underwent transanal endoscopic operations for resection of rectal adenomas, from April 2000 to June 2018. All surgeries and clinical follow-up evaluations were performed by the same surgical team. The two referral centers were the Hospital São Paulo, and Hospital de Clínicas, Faculdade de Medicina de Ribeirão Preto, Universidade de São Paulo (FMRP-USP).

### *Data collection*

A search was performed in the medical charts and then followed and completed a script that was designed with Microsoft® Excel and contained the variables to be determined for the study from April 2000 to June 2018. Inclusion criterion: patients with rectal adenomas subjected to transanal endoscopic surgery. During this period, 99 patients underwent transanal surgery with the proctoscope to treat rectal adenomas. Patients with a previous history of rectal cancer were excluded. The analyzed data included the patient’s clinical history, physical, proctological, and colonoscopic examinations, clinical evolution, operative time, suture type (manual or mechanical), lesion resection (fragmented or nonresected), adenoma characteristics (distance from the anal rhyme, size in centimeters, histological type, and degree of dysplasia), incidence of adenocarcinoma in the surgical specimen, and presence or absence of free margins. Intraoperative complications and length of hospital stay were also evaluated, as well as information on postoperative follow-up, rectal adenoma recurrence, treatment type (endoscopic or surgical), complications, and death.

### *Surgical proctoscope description*

**Figure 1:**
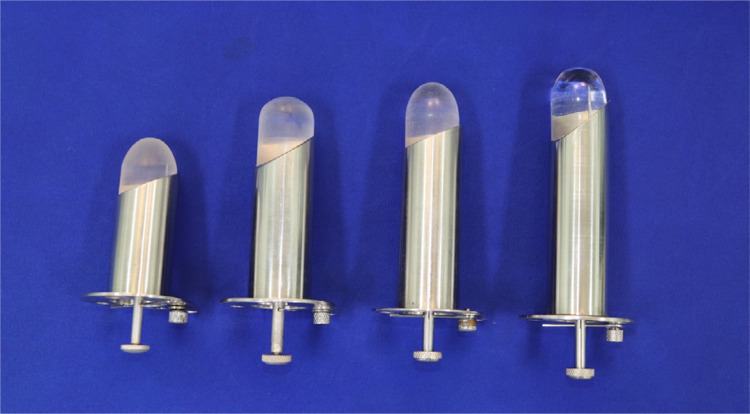
Surgical proctoscopes.

**Figure 2:**
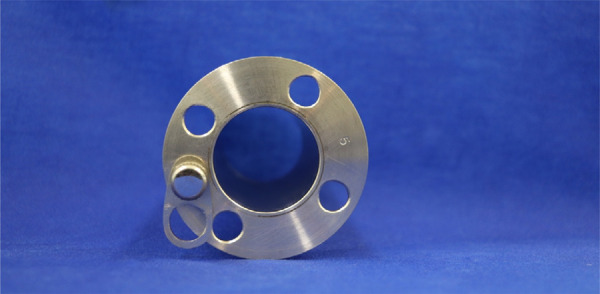
Proctoscope end.

Surgical proctoscopes were devised and constructed at HCFMRP-USP and were previously described in 2008^10,11^. The proctoscopes are made of stainless steel and resemble a cylinder. They have diameters of 4 cm and are 7, 9, 12, or 20 cm long (Fig. 1). At one end, there is a 1-cm-wide tab with four equidistant holes, which are intended to fix the device by sutures to the anal border. In the same tab, there is a small 2-cm-long rectangular device with rounded contours that fixes it to the proctoscope tab (Fig. 2). This device is movable and is positioned so that its perforated end is located in the lumen of the proctoscope. The screw on the other side of the tab is threaded so that the device can be fixed there. This screw serves to fit the end of the light source optical fiber cable, which illuminates the operating field.

**Figure 3:**
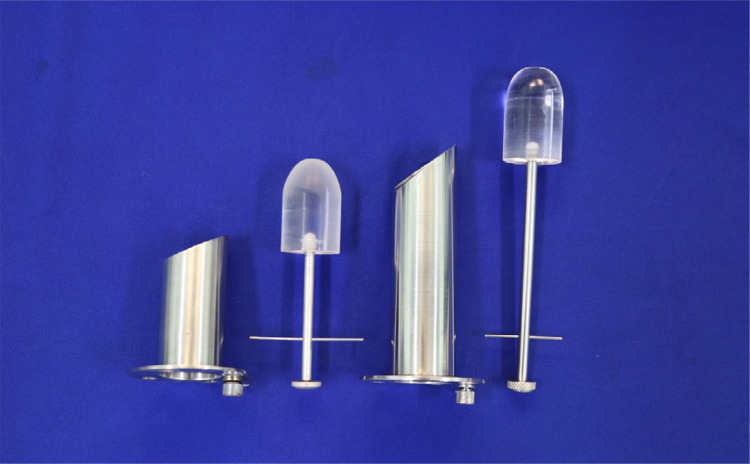
Proctoscope and its mandrel.

**Figure 4:**
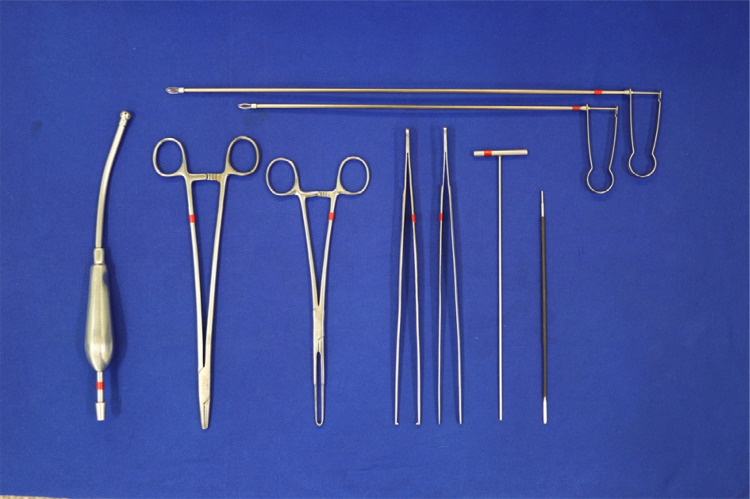
Surgical instruments and accessories.

The other end of the device, which is introduced anally, has a beveled appearance and blunt edges. Because there is no insufflation, this configuration allows the device to be positioned when the lesion is located so that the longest part of this extremity moves the contralateral mucosa away from the lesion and remains in the center of the operative field. This set includes a mandrel, which is placed inside the rectoscope at the time of anal insertion and serves as a dilator and guide for the proctoscope (Fig. 3), and surgical instruments and accessories (Fig. 4).

**Figure 5:**
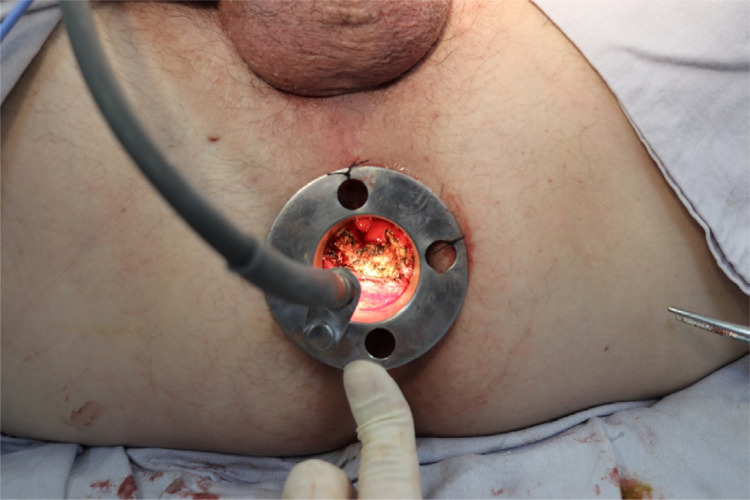
Proctoscope positioned in the anus.

After being positioned in the anus (Fig. 5), the proctoscope has features such as a light source with an optical fiber cable, electrocautery, polypectomy handles, and conventional material such as tweezers and a needle holder. Making a proctoscope with some of its main components approximately costs U$ 300.00 (three hundred dollars).

### *Statistical analysis*

The information was compiled in Microsoft® Excel spreadsheets for descriptive analysis and comparison between the variables by Fisher’s exact test. The results were analyzed with the IBM® SPSS® Statistics 20.0 computer program (Armonk, New York). For all the analyses, the significance level was set at 5%.

## Results

### *Cohort and demographics*

A total of 99 patients who underwent endoscopic transanal surgery with the proctoscope described here were included. The mean age was 65.3 ± 13.3 years, and most patients were male and had ASA 2. The median duration of symptoms was 12 months, with an interquartile range (IQR) of six to 15 months. Of the 99 colonoscopies performed, 43.4% diagnosed other adenomas, in addition to rectal polyps. The main characteristics of the operated patients and their clinical manifestations are shown in Table 1.

**Table 1:** Main characteristics of the studied patients.

Features	Frequency	
	n	%
**Gender**		
Male	56	56.6
Female	43	43.4
ASA*		
(1) no comorbidities	30	30.3
(2) mild systemic disease	53	53.5
(3) severe systemic disease	16	16.2
**Smoking**	22	22.2
**Ethylism**	2	2.0
**Clinical manifestations**		
Mucus	69	53.8
Blood	69	53.8
Diarrhea	38	29.4
Rectal pain	36	28.2
Rectal tenesmus	26	20.5
Weight loss	23	17.9
Anal incontinence	15	11.5
Constipation	5	3.84
Anemia	2	1.28
Asymptomatic	21	21.2
**Synchronic Adenomas**	43	43.4

*ASA: American Society of Anesthesiology Surgical Risk Rating.

### *Operative parameters*

The main characteristics of the operated rectal adenomas were analyzed, such as size, distance from the anal verge, histological type, degree of dysplasia and presence of adenocarcinoma in the anatomopathological region of the surgical specimen and its stage. The main characteristics of the resected adenomas are described in Table 2.

**Table 2:** Main characteristics of the resected adenomas.

Feature	Numbers
Size (mean ± SD*)	4.6 ± 2.3 cm
**Anal Edge Distance (mean ± SD)**	5.6 ± 3.3 cm
**Histological type n (%)**	
Villous tubule	58 (58.6%)
Villous	27 (27.3%)
Tubular	14 (14.1%)
**Degree of dysplasia n (%)**	
High	62 (62.7%)
Low	33 (33.3%)
Moderate	1 (1.0%)
Undetermined	3 (3.0%)
Presence of adenocarcinoma n (%)	22 (22.2%)
**Adenocarcinoma staging n (%)**	
T0 - in situ	13 (59%)
T1 - invasion to submucosa	5 (23%)
T2 - invasion up to own muscle	4 (18%)

The mean operative time was 65.3 ± 41.7 minutes. The surgical specimen was fragmented in 48.5% (48) of the operations. Manual sutures (88.9%, n = 88) and mechanical sutures (11.1%, n = 11) were used to close the surgical wound in the rectum. The conversion rate for open surgery was 3% (n=3): there was an intraoperative accident in which the rectum was perforated in the peritoneal reflection, thus evolving to abdominal rectosigmoidectomy, and there were two more laparotomies due to technical difficulties resulting from the extension of the lesion (10 and 15 cm). The length of hospital stay was 2 ± 1.6 days.

### *Postoperative outcomes and recurrence*

The rate of postoperative complications was 5% (n=5): two anastomosis substenoses, two dehiscences, and one anal incontinence. Readmission to the hospital occurred in 2 patients, both of whom had dehiscence of the anastomosis. One patient needed a temporary stoma. Microscopic margins were described as free in 59 surgeries (59.6%). In the remaining 40 surgeries (40.4%), the margins were compromised or could not be evaluated due to piece fragmentation (p value with statistical significance). The results in Table 3 are described by univariate analysis and their respective p values.

**Table 3:** Analysis of variables associated with the presence of compromised or unknown margin of the resected rectal adenomas.

Features	Compromised/unknown margin		*p*
	N	%	
**Adenoma Size**			
≥ 5cm	20	46.5	0.44
< 5cm	19	35.8	
**Adenoma Height**			
≥ 10cm	6	31.6	0.30
< 10cm	34	42.5	
**Piecemeal**			
Yes	26	54.2	0.008
No	14	27.5	

Nineteen instances of recurrence (19.2%) of the rectal lesion were diagnosed during patient follow-up. The median time until relapse was 16 months (IQR 4-45). The mean follow-up was 80 ± 61.5 months. There were no deaths related to the procedure. The rectal adenoma height and surgical specimen fragmentation were related to lesion recurrence. Of the 19 recurrent lesions, 15 operations demonstrated a fragmented surgical specimen, and of these, 7 were lesions located in the high rectum, 9 were ≥ 5 cm in size, and 13 had high-grade dysplasia. Table 4 presents the results of the univariate analysis with the respective p values.

**Table 4:** Analysis of the variables associated with rectal adenoma recurrence.

Features	Relapse		*p*
	n	%	
**Adenoma Size**			
≥ 5cm	10	23.3	0.43
< 5cm	8	15.1	
**Adenoma Height**			
≥ 10cm	7	36.8	0.04
< 10cm	12	15.0	
**Piecemeal**			
Yes	15	31.2	0.004
No	4	7.8	
**Margin**			
Compromised/Undetermined	10	25.0	0.45
Free	9	15.0	
**Degree of Dysplasia**			
High	14	22.6	0.30
Low / Moderate	5	13.5	
**Adenocarcinoma**			
Yes	5	22.7	0.75
No	14	18.2	

Postoperative complications were related to an adenoma size ≥ 5 cm (p value with statistical significance). Table 5 displays the results of the univariate analysis with the respective p values.

**Table 5:** Analysis of the variables associated with postoperative complications.

Features	Complications		*p*
	n	%	
**Adenoma size**			
≥ 5cm	5	11.6	0.01
< 5cm	0	0	
**Adenoma Height**			
≥ 10cm	5	0	0.58
< 10cm	0	6.2	
**Piecemeal**			
Yes	4	8.3%	0.19
No	1	2%	
**Suture Type**			
Mechanic	1	9.1%	0.45
Manual	4	4.5%	

## Discussion

Our study involved almost one hundred patients operated on by the same transanal technique with the rigid proctoscope. The surgery was performed in an acceptable surgical time, less than 70 minutes on average, with low rates of postoperative complications, low instances of recurrence and no deaths.

One disadvantage of TEM is the visualization of very low tumors. The rigid proctoscope allows the surgeon to easily remove adenomas <5 cm from the anal verge. TEO has a shorter learning curve, similar to our proctoscope. However, a 3D optical system is not used, TEO experience is limited, and studies about this technique are scarce^7,12^. TAMIS is a safe technique with a short learning curve for laparoscopic surgeons already proficient in single-port procedures, and it provides effective oncological outcomes^13^. However, it has limitations in that the rectoscope cannot be mobilized at the injury site, rectal lesions located behind a rectal valve can be more difficult to access and remove, and an assistant is required to hold and manipulate the laparoscope during the surgery. Our proctoscope can be mobilized during the procedure and can remove lesions with low difficulty levels behind the rectal valves.

We found a mean operative time of 65.3 ± 41.7 minutes. Compared to other surgeries, the technique using the proctoscope was associated with a shorter operative time^14-17^. Our study presented a conversion rate for open surgery of 3%, mainly due to technical difficulties resulting from the extension of the lesion (10 and 15 cm). The conversion rates vary from 1% to 13%. Conversion to low anterior resection occurred in 6% of cases due to difficult access to the lesion and lack of progress in another series^14^. Another study showed that in 6.7% of cases, the TEM procedure was discontinued because complete excision could not be completed endoscopically. In this series, the tumor extended up into the anterior wall of the upper rectum, similar to our study^15^. Some authors described a 13% rate of conversion to Park’s transanal technique^18^, mainly due to the proximity of the anal border and the difficulty in maintaining the pneumorectum^17^. This did not occur in our study because there was no need for a pneumorectum in our proctoscope.

Our study showed rates of intraoperative and postoperative complications of 1% and 5%, respectively, which is lower than those described by other techniques^15,18^. Some authors reported that up to 20% of patients experienced postoperative complications^15^, half of them due to postoperative peritonitis due to intra-abdominal perforation. The authors also reported postoperative bleeding, with some cases requiring blood transfusion. Some studies showed no intraoperative complications after surgery; however, the postoperative complication rates were higher than those in our study (9.7%), mainly due to hemorrhage^12^. Another study had a postoperative complication rate of 10%^17^, with complications including urinary retention, bleeding (requiring return to the operating room for urgent treatment) and suture line dehiscence. None of our patients presented with urinary retention or postoperative hemorrhage.

Our study showed a recurrence rate of 19% during the mean follow-up of 80 ± 61.5 months. To our knowledge, our study has the longest postoperative follow-up time after transanal polyp resection. The majority of studies have lower recurrence rates but a shorter median follow-up period. Recurrence is mainly related to compromised margins and can be detected in short- or long-term follow-ups. In our study, the margins were compromised or could not be evaluated due to piece fragmentation in approximately 40% of patients. In a review of 18 studies involving TEM-resected adenomas with a minimum follow-up of 12 months, the relapse rate was 0% to 15%, and relapse predominated in cases of positive or uncertain margin resection^5^, similar to our results. Another study showed that during a median follow-up period of 15 months, two cases of recurrence occurred^12^. Similar results were reported in another study, concluding that histological evaluation of the resected adenoma was an important predictor of recurrence and had the potential to guide follow-up strategies after surgery^19^. In a systematic review of 266 procedures, the authors observed positive margins in 5% of cases, and margins could not be defined due to tissue fragmentation in almost one-third (31%) of the surgical specimens^20^, which was also demonstrated in our study. Of the 19 patients with relapse in our series, the majority (57.9%) underwent a second transanal resection, and 36.8% of patients with recurrent lesions underwent rectosigmoidectomy due to a high adenoma location or cancer.

Another advantage of the proctoscope is with regard to cost. The proctoscope used in this study is inexpensive compared with other technologies. In addition, other techniques, such as TEO, require a learning period^21^. In addition, TEM has not gained wide acceptance in the surgical community and is routinely performed in only a few dedicated centers, mainly because of the long and challenging learning curve, high instrumentation costs, and relatively limited number of patients who are suitable for the procedure^8,22^. The initial cost of specialized TEM equipment is perceived by some surgeons as a limiting factor for the widespread adoption of this technique^23^. The proctoscope used in this study is cheap (approximately U$ 300), and conventional and laparoscopic surgical instruments can be used without the need for gas insufflation, providing a three-dimensional view and allowing greater accessibility for surgeons^10,11,24^.

Our study has several limitations. First, although a prospective database was used, this study was limited by its retrospective nature. However, it has the longest follow-up period to date. Second, this study was limited by the number of patients included, mainly because the surgical indication (large polyps in the rectum) was restricted. Third, the data were heterogeneous with regard to the size and location of the polyps. Therefore, further longitudinal studies using a more representative sample are needed to analyze the outcomes among patients with the same kind of polyps or similar disease stages. However, our series included only patients with rectal adenomas, which can compensate for this disparity in relation to the size and location of polyps. Finally, we compared our results with those of other techniques, even with the same study population (rectal adenoma patients). It is difficult to compare different transanal techniques, mainly due to the retrospective nature, with heterogeneous groups and many indications, in many studies.

## Conclusions

In summary, our study demonstrated that surgery to remove rectal adenomas using a rigid proctoscope is feasible and safe, with low rates of intraoperative accidents and postoperative complications, a shorter operative time. In addition, it requires inexpensive equipment and conventional surgical instruments, dismisses the need for gas insufflation, and allows greater accessibility for surgeons. This proctoscope has been proven to be a viable and more accessible technique, which allows its implementation and widespread use in surgical practice.
